# Sexually transmitted infections in polygamous mating systems

**DOI:** 10.1098/rstb.2012.0048

**Published:** 2013-03-05

**Authors:** Ben Ashby, Sunetra Gupta

**Affiliations:** Department of Zoology, University of Oxford, Oxford OX1 3PS, UK

**Keywords:** sexually transmitted infections, host heterogeneity, sexual contact network, polygamy, evolution of virulence, mating strategy

## Abstract

Sexually transmitted infections (STIs) are often associated with chronic diseases and can have severe impacts on host reproductive success. For airborne or socially transmitted pathogens, patterns of contact by which the infection spreads tend to be dispersed and each contact may be of very short duration. By contrast, the transmission pathways for STIs are usually characterized by repeated contacts with a small subset of the population. Here we review how heterogeneity in sexual contact patterns can influence epidemiological dynamics, and present a simple model of polygyny/polyandry to illustrate the impact of biased mating systems on disease incidence and pathogen virulence.

## 1. Introduction

Evidence from anthropological and ethological studies suggests that there is much heterogeneity in sexual behaviour of humans and animals [1–4], both in rates of sexual activity and in patterns of sexual contact. Polygynous and polyandrous mating systems are particular examples, where one sex tends to have a much higher variance in partner acquisition rate compared with the other sex. It is well-established that the structure of a mating system can have a profound influence on genetic diversity [5] and the evolution of sexually selected traits [6]; here we discuss how such heterogeneities can influence epidemiological dynamics of sexually transmitted infections (STIs) and the evolution of associated pathogens.

We begin by reviewing how host heterogeneity in sexual behaviour can influence the epidemiological dynamics of STIs, mainly in the context of human diseases, such as HIV-1 and gonorrhoea. We then introduce an individual-based model for biased (polygynous and polyandrous) mating systems, where movement is based on perception of reproductive failure. In line with the results of Thrall *et al.* [7], we observe that the polygamous sex exhibits much lower levels of infection than the monogamous sex and the difference tends to increase with greater variance in attractiveness; in addition, we show that the difference between the polygynous and polyandrous scenarios depends on the probability of sterility. Finally, we present an example of how the evolution of pathogen virulence can be explored within this framework by introducing a simple dichotomy in the trade-off between transmissibility and duration of infection. Within our system, the less virulent pathogen tends to be favoured for high degrees of polygamy, demonstrating a clear link between mating patterns and pathogen evolution.

## 2. Influence of sexual contact patterns on epidemiology of sexually transmitted infections

From an epidemiological point of view, the transmission dynamics of STIs are fundamentally different to those of many other infectious diseases. First, sexual contact rates are usually invariant to population size, which means that there is no critical population density required for a typical STI to persist. By contrast, the rate at which non-STIs spread is often dependent on the density of the host population [8]. Second, there is often considerable variation in sexual behaviour both within [4] and between [9] populations. Highly active members of the population (e.g. sex workers in human populations, alpha males in animal populations) will generally be at much greater risk of receiving and transmitting an infection than monogamous couples and so contribute disproportionately to the spread of disease as well as representing important targets for disease control.

In order to establish how heterogeneity in sexual behaviour can alter epidemiological dynamics, it is first useful to define the basic reproductive number, *R*_0_, of an infectious agent in a well-mixed population:
2.1
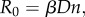



where **β** is the probability of transmission per contact, *D* the average infectious period and *n* the average number of contacts (see [8] for a more detailed discussion of *R*_0_). The basic reproductive number is essentially the average number of secondary infections that a single infectious individual will produce in an entirely susceptible population. Hence the infection will tend to spread if *R*_0_ > 1, but will go extinct if *R*_0_ < 1. This formulation of *R*_0_ is based on an idealized, randomly mixing homogeneous population, but real mixing patterns are likely to be more complex due to spatial constraints and variations in host behaviour. If we imagine a continuum with well-mixed and highly structured populations at the extremes, then most real populations will fall somewhere between the two. Note that the position of a population on this continuum is dependent on the transmission pathways of a particular infection; a population may be relatively well-mixed in terms of social contacts, but might demonstrate a high degree of heterogeneity in sexual mixing patterns.

In general, casual contacts between humans tend to be ephemeral and non-repetitive, whereas sexual contacts are more stable [10]. In addition, variation in close contact rates is likely to be much smaller than variation in sexual partner acquisition rates. For example, Mossong *et al.* [11] found that adolescents had just over twice the number of close contacts than the elderly, but studies of sexual mixing patterns generally find power-law distributions in partner acquisition rates, sometimes ranging over three orders of magnitude [4,12]. Power-law distributions are also likely to be applicable to a variety of animal mating systems, particularly where a few members of one sex are dominant (i.e. polyandry or polygyny). Hence, the above formulation of *R*_0_ may be a reasonably good indicator of epidemic spread for infections transmitted by close contact, but is likely to be a poor approximation for STIs. In addition, sexual contacts tend to be much less frequent than social contacts, lowering the value of *R*_0_. Hence, one explanation as to why many STIs are associated with chronic, asymptomatic diseases is that this increases the value of *D* to compensate for lower contact rates.

**Figure 1.**
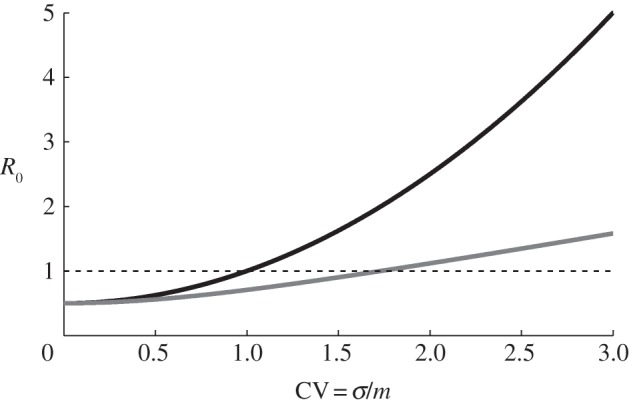
The relationship between the coefficient of variation, CV = **σ**/*m*, of the partner acquisition rate distribution and the basic reproductive number, *R*_0_, for fixed values of **β**, *D* and *m*. (**β** = 0.1, *D* = 5 year and *m* = 1 yr^−1^, so that **β*Dm* = 0.5). The black curve shows the relationship when there is no distinction between the sexes (*R*_0_ = **β*Dm*(1 + CV^2^)) and the grey curve shows the relationship when variation only occurs in one sex (*R*_0_ = **β*Dm*(1 + CV^2^)^1/2^). The dotted line corresponds to the threshold for an epidemic (*R*_0_ > 1). Adapted from May *et al.* [15].

Sexual transmission can be considered part of a much broader class of models with heterogeneous contact rates, usually referred to as ‘super-spreader’ models, where a few members of the population have a disproportionately large effect on disease spread [8,12]. For super-spreader models, we can incorporate this heterogeneity into the formula for *R*_0_ by compartmentalizing the population according to contact rates, so that *N_i_* is the proportion of the population that acquires *i* contacts per unit time [13,14]. Retaining the assumption that mixing is random, this allows us to calculate an effective contact rate, *c*, over the distribution:
2.2
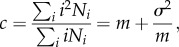

where *m* and **σ**^2^ are the mean and variance in contact rates, respectively [14]. The formula for the basic reproductive number now becomes *R*_0_ = **β*Dc*; clearly, any heterogeneity in contact rates will increase the value of *R*_0_ and hence the initial growth rate of the epidemic (figure 1).

In the context of sexual transmission, the second formulation of *R*_0_ applies to a homosexual population, but it can be readily generalized for a heterosexual population by separating the effective partner acquisition (i.e. contact) rate into male (*c*_m_) and female (*c*_f_) components. If we also assume that there are differential transmission rates across the sexes (as is common with many STIs), then our equation for *R*_0_ becomes:
2.3
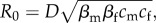

where **β**_m_ is the transmission rate from males to females and **β**_f_ is the transmission rate from females to males [8,16]. If *c*_m_ = *c*_f_ then *R*_0_ will asymptote towards quadratic growth with the coefficient of variation (CV = **σ**/*m*), but if variation is limited to one sex then *R*_0_ will tend towards linear growth. Changes in the variance will be most significant when *R*_0_ is close to unity (the epidemic threshold), as relatively small changes in the size or the behaviour of the core group can determine whether an epidemic will occur (figure 1).

The effective partner acquisition rates can also be used to estimate the ratio of cases in males (*C*_m_) to females (*C*_f_) during the early stages of the epidemic
:2.4
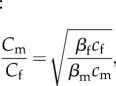



(see [8], §11.3.9 for a more detailed discussion; also [15]). This work was originally motivated by the spread of HIV in Africa, but the principles can be applied to other populations that exhibit host heterogeneity. For example, the ratio *C*_m_/*C*_f_ suggests that the dominant sex in a biased mating system (e.g. males in polygynous systems) will tend to exhibit lower than average levels of infection, although this could be counterbalanced by differences in transmission probabilities. Indeed, it is thought that *C*_m_/*C*_f_ ≈ 1 for HIV-1 in many parts of Africa because the partner acquisition rates (*c*_f_ > *c*_m_) are more or less balanced by differences in transmission rates (**β**_f_ < **β**_m_) [8,16]. Note that even if *C*_m_/*C*_f_ ≈ 1, the distribution of infection will still be biased towards more sexually active members of the population.

Further complications will arise if the population does not mix homogeneously, for example where people tend to show a preference for mixing with similar individuals (assortativity). Mixing patterns have been found to vary considerably between human populations, ranging from highly assortative [17], to highly disassortative (i.e. showing preference for dissimilar individuals) [18] mixing. The degree of assortative mixing may also vary within a population: for example, Wylie & Jolie [19] found that assortative mixing was common in linear components of a sexual contact network (SCN), but disassortative mixing was common in radial components.

**Figure 2.**
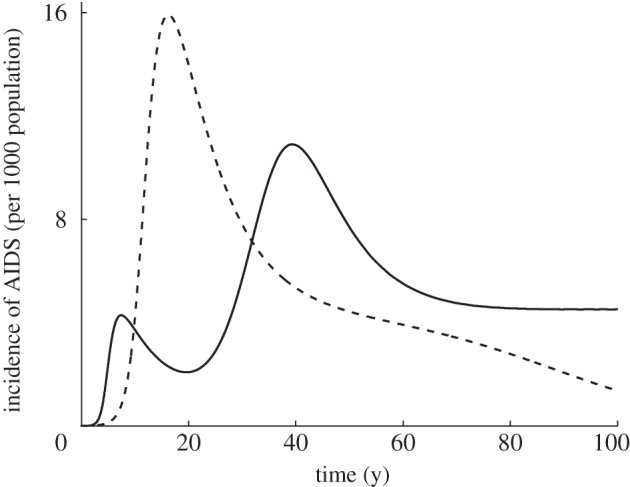
Incidence of AIDS as a proportion of population size in populations that exhibit highly assortative (solid curve) and highly disassortative (dotted curve) mixing. Highly assortative (solid line) mixing tends to lead to rapid growth during the early stages of an epidemic and can produce multiple peaks in disease incidence. Highly disassortative (dashed line) mixing is usually characterized by slower initial growth, but a higher peak in the incidence of AIDS. The model is adapted from Gupta *et al.* [21] (see the electronic supplementary material).

In order to model heterogeneous mixing, we can group individuals according to their level of sexual activity (i.e. partner acquisition rate) and describe interactions between groups using a ‘mixing-matrix’ [13,14,16,20,21]. A simple mixing-matrix for a population split into high (H) and low (L) activity groups would be
2.5
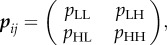

where *p_ij_* is the proportion of sexual contacts that individuals from group *i* make with members of group *j*. For completely assortative mixing, ***p****_ij_* is equal to the identity matrix (*p_ii_* = 1, *p_ij_* = 0 for *i*≠*j*). There is usually no single disassortative extreme, however, as disassortativity is maximized whenever the elements of the main diagonal of ***p****_ij_* are minimized [21]. For a given mixing matrix, we can measure the degree of assortativity, *Q*, in the population as
2.6
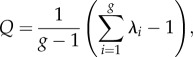

where *g* is the number of activity groups and **λ*_i_* are the eigenvalues of ***p****_ij_*. Gupta *et al.* [21] found that highly assortative mixing (*Q* ≈ 1) tends to lead to more rapid epidemic growth and can produce multiple peaks in disease incidence. By contrast, highly disassortative mixing (*Q* ≈ −1/(*g* − 1)) is generally associated with slower epidemic growth, but will typically produce higher peaks in disease incidence (figure 2). This method highlights the importance of host heterogeneity in the spread of STIs and suggests that targeting control measures at the core group is optimal, although the efficacy of such procedures will depend on the size of this group and the degree of assortative mixing in the population.

While this approach is a useful way of capturing host heterogeneity, it cannot capture some of the complex interactions found in real populations that are imposed by other factors than level of sexual activity. Such mean-field approaches assume that sexual activity classes are well mixed so that if an infectious individual mixes with a particular activity class, then all members of that class will have an equally increased risk of infection. In reality, the risk of infection will be limited to those who have sexual contact with the infectious individual rather than the entire activity class. An alternative method is to use an SCN which captures heterogeneity at the level of individuals and provides a means of replicating more realistic transmission pathways. This approach is particularly well suited to STIs, as transmission pathways are usually much more clearly defined (i.e. sexual contact) than for non-STIs. However, there are many problems associated with collecting data on real SCNs, including biases in reporting and difficulties with linking up components in a larger network [17,22], although some attempts have been made for small populations [18,19,23–26].

Mean-field models and SCNs may show good agreement over the main part of an epidemic, but fundamental differences in structure are likely to have significant consequences for the spread of infection during the early stages [27]. Keeling [27] showed that in a general SCN, the probability that an index case *i* will fail to pass on the infection to any of their *n_i_* contacts is
2.7
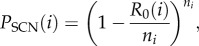

where *R*_0_(*i*) = min(**β*Dn_i_*,*n*) is the expected number of secondary infections to be caused by the index case. The corresponding probability of extinction after the first generation using a mean-field approximation is:
2.8
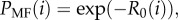

which satisfies *P*_MF_(*i*) > *P*_SCN_(*i*). Hence the probability of extinction during the first generation is always higher in a mean-field approximation than it is in the corresponding SCN, but as the number of contacts increases the two values converge (i.e. 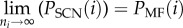). Averaging these values over the entire population gives the probability that a randomly introduced infection will die out after the first generation. Note that in real populations, an index case is probably more likely to occur among highly active individuals, which will decrease the probability of extinction during the first generation.

One advantage that SCNs have over mean-field approximations is their ability to capture long-term partnerships that are commonly found in many human and animal populations. In particular, serially monogamous partnerships (common among birds as well as humans) cannot be modelled using traditional mean-field approaches. Computer-generated contact networks can be used to recreate mixing patterns observed in real populations [25], by connecting individuals (nodes) to other members of the population preferentially, based on factors such as proximity, cluster size or assortativity. Studies of simulated epidemics on SCNs have revealed that concurrent partnerships are crucially important to the spread of many STIs [28–30]. For example, Morris & Kretzschmar [29] demonstrated that the size of an epidemic grows exponentially with the relative number of concurrent partnerships in a population. Reducing the number of concurrent partnerships in a population is therefore likely to be an effective mechanism of disease control.

## 3. Exploring the role of mating system structure on epidemiological dynamics

We now introduce a simple SCN model to illustrate how epidemiological dynamics of STIs can be influenced by biased (i.e. polygynous or polyandrous) mating systems. Our model is similar to that of Thrall *et al.* [7], which was used to explore how disease prevalence is affected in a general biased mating system with random movement between mating groups. The authors found that disease prevalence in the two sexes tends to diverge as variance in mating success increases and that less attractive members of the population may have higher lifetime reproductive success in the presence of a sterilizing STI. We build on this study by varying the probability that an infection will cause host sterility and by basing movement decisions between mating groups on an individual's perception of reproductive failure. This simple stay-or-stray decision introduces behavioural differences between polygynous and polyandrous mating systems, as females are generally better placed to infer their reproductive success. Given that a range of complex mate choice behaviour has been observed, including the avoidance of parasitism, inbreeding and harassment (see [31] for a review of mate choice behaviour), it seems reasonable that a simple binary decision of prior reproductive success or failure could influence mate choice. In fact, a meta-analysis of mate fidelity among 35 species of monogamous birds found that divorce rates were significantly higher among unsuccessful than successful pairs [32], providing strong evidence that prior reproductive failure can reduce mate fidelity.

We consider a population of constant size, composed of *N*_m_ males and *N*_f_ females, where one sex is polygamous and the other is serially monogamous. We follow Thrall *et al.* [7] in assigning members of the serially monogamous sex to mating groups consisting of a single member of the polygamous sex. Each polygamous individual, *i*, is assigned a fixed level of attractiveness, *a*(*i*), according to a power-law distribution with shape parameter α
3.1
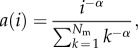

with 
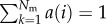
. Members of the polygamous sex are then assigned non-overlapping line segments, *L*(*i*), with lengths equal to their attractiveness:
3.2
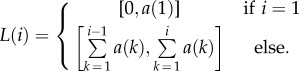



**Figure 3.**
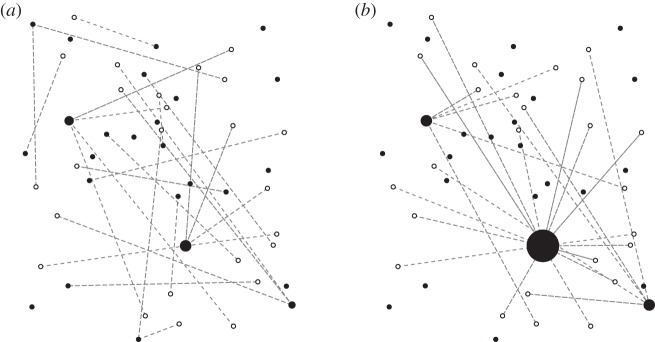
Example sexual contact networks (SCNs) in a biased mating system. Sexual contacts are indicated by dotted lines connecting the serially monogamous sex (empty circles) to the polygamous sex (filled circles). Members of the polygamous sex attract mates based on their relative ‘attractiveness’ (represented here by circle size), which is based on a power-law distribution (equation (3.1)) with (*a*) **α** = 1 and (*b*) **α** = 2. (*a*) Lower values of **α** produce more balanced SCNs, with less variation in the number of partners. (*b*) Higher values of **α** cause a few members of the population to dominate the network and leave many members of the polygamous sex without partners.

For each serially monogamous individual *j*, a random number, *r*(*j*) *ε* (0,1), is then generated. The connections between males and females are given by the adjacency matrix ***A****_ij_*, where *A_ij_* = 1 if *r*(*j*) *ε* *L*(*i*) and is zero otherwise. For small values of **α**, there is little variation in attractiveness and so the network approaches serial monogamy for both sexes, although some concurrent partnerships may still occur (figure 3*a*). For large values of **α**, the network is dominated by a single polygamous individual who is connected to a large proportion of the serially monogamous population (figure 3*b*). We refer to these scenarios as having low and high degrees of polygamy, respectively.

A randomly chosen member of the monogamous sex initiates the epidemic, after which susceptible individuals are infected with probability **β*I*, where **β** is the probability of transmission per contact and *I* is the total number of infectious contacts for a given individual. For members of the polygamous sex, the number of infectious contacts will be equal to the total number of infected individuals in their mating group, whereas for the monogamous sex *I* = 1 if their mate is infected and is zero otherwise. We also include an external force of infection, such that susceptible individuals are randomly infected with probability **κ**. Individuals recover from infection with probability **σ**, at which point they become susceptible to infection again. Infection causes no increase in mortality, but does carry a risk of permanent sterility (probability γ).

We assume that serially monogamous individuals stay within a mating group unless they are certain that they have not successfully produced offspring in the previous mating season. For the purposes of our model, we assume that mating is only successful provided both partners are not sterile. If they are certain that they have been unsuccessful, then they will reassess their mate choice with probability 1 – **ρ**, where **ρ** is inertia to switching mating groups. In real populations, searching for a new mate may be risky (e.g. increased chance of predation, exposure to new pathogens or risk of exclusion; [31]) and so it is reasonable to assume that the serially monogamous sex will only leave a mating group if they are certain that they have not successfully reproduced. If an individual leaves a mating group, then they are immediately reassigned to a new mating group according to the original procedure described above. Deaths occur randomly with probability **μ**; we keep the population size constant by assuming that there is either always a surplus of offspring, or that the immigration rate is sufficiently high to maintain this balance.

The description of polyandrous and polygynous mating systems has been identical up until this point, but they can be distinguished by the behaviour of sterile members of the monogamous sex. In a polygynous system, each female is able to independently determine whether she has successfully reproduced or not. If she has been unsuccessful, then she may opt to choose an alternative mate in future. In a polyandrous system, however, success is based on the ability of the sole female in the mating group to reproduce. As long as there is a chance that a male has successfully reproduced (i.e. the female and at least one male in the group are both fertile), then the benefits of staying within a mating group may outweigh the costs of leaving, even if males are unable to detect who is the father of an infant.

**Figure 4.**
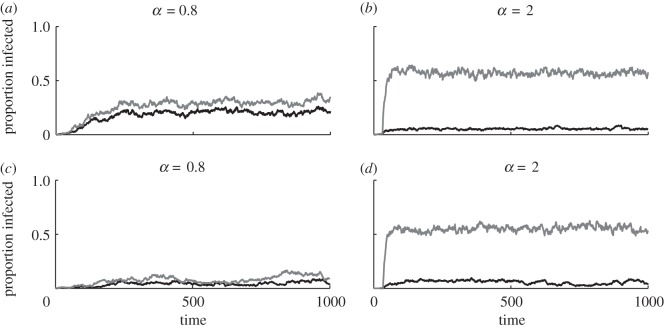
Dynamics of an SIS-type infection on SCNs for varying degrees of polygamy (**α**) and movement probabilities (**ρ**). (*a*,*b*) Are examples from a polygynous species, whereas (*c*,*d*) are examples from a polyandrous species. Higher values of **α** correspond to higher variation in the number of partners for the polygamous sex (equation (3.1)). Infection is usually more prevalent in the serially monogamous sex (grey) than in the polygamous sex (black) (parameters: *N*_m_ = *N*_f_ = 500; **β** = 0.1; γ = 0.5; κ = 10^−4^; **μ** = 0.01; **ρ** = 0.5; **σ** = 0.05).

**Figure 5.**
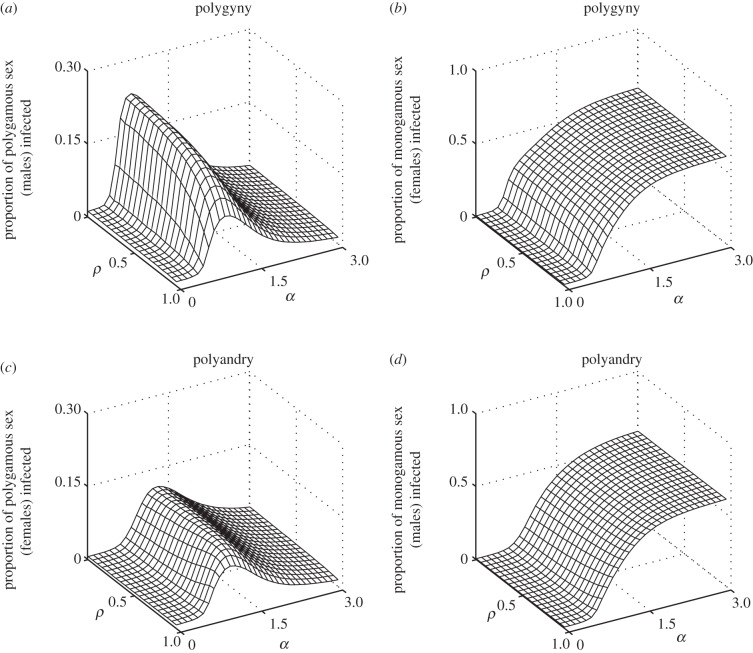
The average proportion of the (*a,c*) polygamous sex and (*b,d*) the monogamous sex that are infected for varying degrees of polygamy (**α**) and inertia to switching mating groups (**ρ**). (*a*,*b*) Data from a polygynous system; (*c*,*d*) data from a polyandrous system. Higher values of **α** correspond to higher variation in the number of partners for the polygamous sex (equation (3.1)). For the monogamous sex, the prevalence of infection tends to increase with higher degrees of polygamy and lower inertia. By contrast, the prevalence of infection in the polygamous sex peaks at intermediate values of **α**. This peak is higher for polygyny than for polyandry (parameters: *N*_m_ = *N*_f_ = 500; **β** = 0.1; **γ** = 0.5; **κ** = 10^−4^; **μ** = 0.01; **σ** = 0.05).

Figure 4 shows example simulation dynamics for various degrees of polygamy (**α**) in polygynous (figure 4*a,b*) and polyandrous (figure 4*c,d*) mating systems. For relatively low values of α, the infection is able to spread to a reasonably large proportion of a polygynous population, but it is unable to spread extensively in a polyandrous population. For higher values of **α**, the infection is able to propagate through both polygynous and polyandrous populations, with little difference between the two scenarios. At this extreme, most of the monogamous population mates with a small set of individuals, leaving the rest of the polygamous sex disconnected from the network. This simultaneously increases the average exposure of the monogamous sex to infection, while decreasing the average exposure of the polygamous sex. As in Thrall *et al.* [7], we observe that the polygamous sex tends to exhibit much lower levels of infection than the monogamous sex and the difference tends to increase with greater variance in attractiveness. This is further emphasized in figure 5, where the average prevalence of infection in the monogamous sex generally increases with larger values of **α**, but peaks at intermediate values of **α** for the polygamous sex.

The number of cases in the monogamous sex is largely invariant to changes in the level of **ρ** (inertia); this is true for both polygynous and polyandrous scenarios (figure 5*b,d*). Similarly, the prevalence of infection in polyandrous females is only marginally influenced by **ρ** (figure 5*c*). In accordance with Thrall *et al.* [7], the average prevalence of infection in polygynous males decreases with greater inertia, but only for intermediate values of **α** (figure 5*a*). In fact, the average number of males infected in a highly mobile population (**ρ** = 0) is approximately double that when movement is more limited (**ρ** = 0.9). Lower values of **ρ** can lead to more mixing between groups and increases mating opportunities for less attractive members of the polygamous sex. Hence, equilibrium levels of infection tend to increase with lower inertia, but the polygamous sex is disproportionately affected. Mixing tends to be much less common in our polyandrous system, as males are less able to determine whether or not they have successfully produced offspring and so generally choose to stay in a mating group. The opposite is true in the polygynous scenario, as females are always able to distinguish success from failure. This may well be a double-edged sword: although polyandry may restrict movement and limit the spread of infection within the population as a whole, it may increase infection locally.

**Figure 6.**
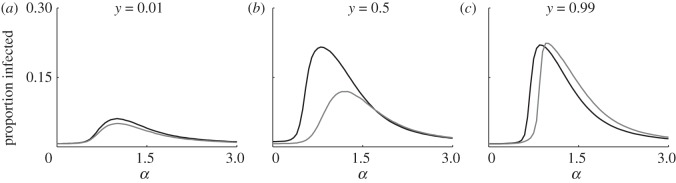
The average proportion of the polygamous sex that is infected for polygynous (black) and polyandrous (grey) mating systems. The difference in epidemic size between the two mating systems peaks at intermediate values of **α** (degree of polygamy) and **γ** (probability of sterilization) (parameters: *N*_m_ = *N*_f_ = 500; **β** = 0.1; **κ** = 10^−4^; **μ** = 0.01; **ρ** = 0.5; **σ** = 0.05).

We find that the probability of sterility (**γ**) is also an important factor in determining disease prevalence when movement between groups is based on reproductive failure. In particular, the difference between the polygynous and polyandrous scenarios was found to be maximized for intermediate values of **γ**, but only for low to intermediate values of **α** (figure 6).

**Figure 7.**
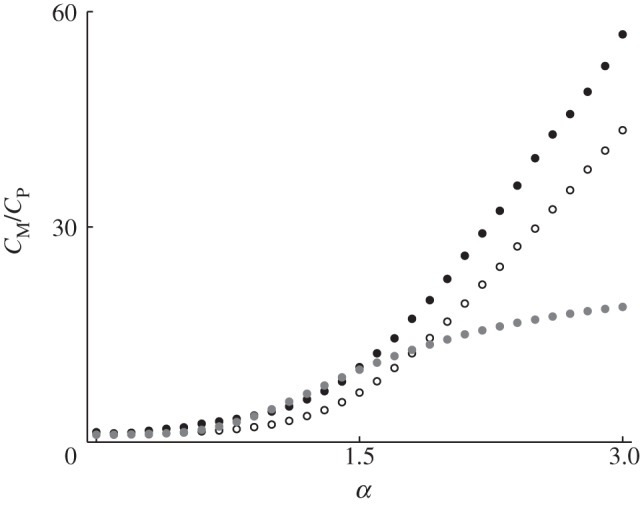
The ratio of cases in the monogamous sex (*C*_M_) to the polygamous sex (*C*_P_) during the early stages of the epidemic for varying degrees of polygamy (**α**). Empty circles correspond to a polygynous system, filled black circles correspond to a polyandrous system and grey circles correspond to the predicted ratio (as per equation (2.4)). The ratio *C*_M_/*C*_P_ is generally higher in polyandrous systems than in polygynous systems. For low to moderate values of **α**, the prediction and actual ratios are generally in good agreement, but this breaks down as **α** increases.

In §2, we discussed how the ratio of male to female cases during the early stages of an epidemic could be predicted based on the transmission rates between sexes and partner acquisition rates (equation (2.4)). Figure 7 compares this prediction (using attractiveness, *a*(*i*), as a proxy for partner acquisition rates) with the actual ratio of cases between the monogamous sex (*C*_M_) and the polygamous sex (*C*_P_) for our model. It is clear that the polyandrous system tends to have a greater bias towards infection in the monogamous sex compared with polygynous systems. As discussed earlier, this is due to increased mixing in polygynous systems exposed to a sterilizing pathogen. For low to moderate degrees of polygamy (**α** < 1.5), there is very good agreement between the predicted and actual ratios for the polyandrous scenario, but is generally an overestimate for the polygynous scenario. For higher degrees of polygamy (**α** > 1.5), the predicted and actual ratios tend to diverge, with the prediction increasingly underestimating the actual ratios (for the predicted values, the variance in attractiveness grows linearly with α, giving *C*_M_/*C*_P_ ∼ *O*(√**α**)); this is because the network is increasingly dominated by a very small number of individuals who are in contact with almost the entire monogamous population (i.e. highly disassortative mating).

Thus far, we have only been concerned with the epidemiological dynamics of our model. We now introduce a second pathogen strain into our model in order to consider the evolutionary implications for pathogens in biased mating systems. Each pathogen strain, *p*, has a transmission probability per contact **β*_p_* and recovery rate **σ*_p_*, and it is assumed that there is a trade-off between these two values, such that **β*_p_* = *r*(**σ*_p_* + **μ**)^*s*^ with *r*, *s* > 0 parameters describing the trade-off and **μ** equal to the natural death rate. For human populations, the trade-off for more transmissible strains could be interpreted as an increased likelihood of seeking medical treatment due to more visible signs of disease. For simplicity, we do not allow co-infection to occur: if an individual is challenged by two different pathogens in a single time-step, then one pathogen is randomly chosen to establish an infection. Both strains are introduced at the start of each simulation and susceptible individuals are infected with probability **β*_p_I* + κ, where **β*_p_* is the probability of transmission per contact for pathogen *p*, *I* the total number of infectious contacts for a given individual and **κ** the external force of infection, as before. We assume that the two strains are equally likely to cause sterility.

**Figure 8.**
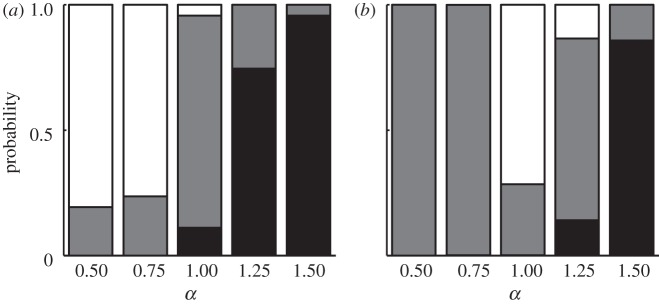
Probabilities of different outcomes when two strains compete, with varying degrees of polygamy (**α**) for (*a*) polygynous and (*b*) polyandrous mating systems. Black bars correspond to strain 1 (less virulent) dominating, white bars to strain 2 (more virulent) dominating and grey bars to coexistence. A strain is defined to be dominating if it accounts for at least 95% of infections during the final 20% of a simulation (parameters: *N*_m_ = *N*_f_ = 500; *r* = 1.5; s = 1.1; **β**_1_ ≈ 0.07; *β*_2_ ≈ 0.56; γ = 0.5; *κ* = 10^−4^; *μ* = 0.01; *ρ* = 0.5; *σ*_1_ = 0.05; *σ*_2_ = 0.4).

Figure 8 shows the probability that each strain will account for at least 95 per cent of infections for various degrees of polygamy (**α**) in the polygynous and polyandrous scenarios when the trade-off between transmission probability per contact (**β*_p_*) and recovery rate (**σ*_p_*) is superlinear (*s* = 1.1). Under polygyny (figure 8*a*), the less virulent pathogen (strain 1) tends to dominate for **α** > 1, the more virulent pathogen (strain 2) dominates when **α** < 1 and coexistence is most common when **α** = 1. The pattern is similar for polyandry (figure 8*b*), but for **α** < 1 neither strain is able to become widely established, allowing both strains to coexist at low levels. For linear and sublinear trade-offs, the less virulent pathogen tends to dominate, but coexistence is often still possible (see the electronic supplementary material, figures S1 and S2).

A possible reason that the less virulent pathogen tended to be favoured for high degrees of polygamy is that maintaining infection in the dominant member of the mating group is likely to significantly contribute to the survival of a particular strain. Hence long infectious periods (low *σ*) are likely to be favoured when the degree of polygamy is high, even if this results in a lower basic reproductive number (i.e. if *s* > 1). By contrast, a higher transmission rate may be favoured when the distribution of partners is much more even (small *α*), as maintaining infection in the most dominant individual in the population becomes less important.

Typically, when multiple pathogens compete for the same pool of hosts, it is predicted that the pathogen with the highest basic reproductive number (*R*_0_) will drive all others to extinction [8]. Host heterogeneity can complicate this picture for two reasons. Firstly, different behavioural patterns between groups will select for different traits in pathogens [33]. Secondly, components of real SCNs are likely to remain unconnected for long periods of time, owing to a combination of spatial constraints, assortative mixing and serial monogamy. Distinct components may increase divergent selection owing to behavioural differences, but they may also provide spatial refugia for less competitive strains to persist.

## 4. Discussion

Host heterogeneity is known to play an important role in the epidemiological dynamics of many infectious diseases, but is particularly relevant to STIs due to the potential for high variability in partner acquisition rates and sexual mixing patterns. High levels of host heterogeneity are usually found in biased mating systems, where large variance in mating success for one sex may leave many individuals isolated from the SCN. Such isolation has been observed in a variety of polygynous and polyandrous mating systems. For example, observations of elephant seals (*Mirounga angustirostris*) indicate that two-thirds of males do not mate during a breeding season and that a high proportion of males are unlikely to breed at all during their lifetime [34]. Similarly, approximately 75 per cent of matings in the insect *Zorotypus gurneui* are carried out by dominant males, leaving most males with only sporadic opportunities for mating [35]. Other mating systems may contain groups of males and females where mating opportunities are limited for subordinate members of the group, as observed in tamarins (*Saguinus*) [36] and white-winged trumpeters (*Psophia leucoptera*) [37]; in both cases, subordinate females are much less likely to copulate than the dominant female. Even in the absence of a strong dominance hierarchy, populations may still exhibit high variance in partner acquisition rates between the sexes. This is particularly evident among human populations in parts of Central, Eastern and Southern Africa, where male migrant workers are often separated from stable female partners for long periods of time and are concentrated in populations with highly unbalanced sex ratios [38]. These factors, along with high unemployment levels for young women, are thought to be responsible for the presence of large numbers of casual sex workers [38]. For these populations, the distribution of partner acquisition rates for males is usually characterized by a high mean and low variance, whereas the distribution for females will have a low mean and high variance.

In general, large skews in risk and reward for level of sexual activity will lead to high levels of host heterogeneity and will be conducive to the evolution of mate choice. Similarly, the complex decisions that govern mate choice and movement between mating groups are likely to be of crucial importance for the evolution of virulence in STIs. Although the extension of our model to investigate pathogen competition was fairly simplistic, it lends some credence to the notion of coevolution between mating systems and STIs. In particular, the finding that less virulent strains with long infectious periods may be favoured in highly skewed mating systems provides an interesting contrast to studies that have found virulence to increase with greater potential for pathogen dispersal [33,39,40].

We made the assumption that individuals are generally averse to leaving a mating group, and will only do so if they are certain that they have not produced offspring. Inertia to change could be due to a number of social and environmental factors, such as competition for mates, exposure to new pathogens, increased predation risk or time and energy costs of mate searching [31]. Conversely, we could have assumed that individuals only stay within a mating group if they are certain of their success. In this alternative scenario, we would expect levels of infection to be generally higher under polyandry than under polygyny.

We based movement between mating groups on a binary decision (perception of reproductive success/failure), but real systems are likely to demonstrate more complex decisions with regard to mate choice. For example, female fur seals (*Arctocephalus gazelle*) show preference for unrelated, heterozygous males, even if this choice requires increased movement [41], and monogamous bird pairs may still divorce even if they have successfully reared young together, indicating that other factors contribute to mate choice [32]. Still, one might speculate that by tending to cause sterility rather than mortality [42], sexually transmitted pathogens could increase divorce rates and movement between mating groups, thereby leading to a higher incidence of disease.

In our model, we chose to hold the host population size constant, so as to keep the degree of polygamy (i.e. the distribution of *a*(*i*), equation (3.1)) fixed and to ensure that any variation in epidemiological dynamics could be unequivocally ascribed to behavioural differences between mating systems. However, it is possible that widespread sterilization could put host populations under considerable pressure: for example, it has been suggested that sterilizing *Chlamydia psittaci* infections may have contributed to declines in koala (*Phascolarctos cinereus*) populations [43]. Similarly, parasitic nematodes (*Trichostrongylus tenuis*) can reduce host fecundity and are believed to be the primary cause of population crashes among red grouse (*Lagopus lagopus scoticus*) [44]. Thus, it is probable that sterilizing infections could lead to counter-adaptations in the host, such as resistance, tolerance and more advanced mate inspection.

It is widely believed that bright plumage and other sexually selected ornaments could be indicators of resistance to parasitism, so that high-quality mates can be readily identified [45]. In proposing the ‘good-genes’ theory, Hamilton and Zuk avoid the lek paradox (the depletion of genetic variation) by arguing that genetic variation can be maintained by parasite counter-adaptation, resulting in coevolutionary cycling [45]. An alternative resolution to the lek paradox is the pathogen avoidance argument, which suggests that there is a trade-off between obtaining high-quality genes (maximizing the fitness of offspring) and risking exposure to infection (potential reproductive failure) [7,46–49]. Neither theory is likely to be universally applicable [50], but it is conceivable that the presence or the absence of virulent STIs could be used to distinguish between the two in some circumstances. STIs are highly relevant to the pathogen avoidance theory, but are less important to the good-genes theory [45] due to the tendency for STIs to be asymptomatic [42]. As such, one might expect to witness the good-genes theory in action if STIs result in obvious host deterioration, are uncommon or are avirulent, but pathogen avoidance behaviour should be more conspicuous among species with virulent, less visible STIs.

## 5. Future directions

Mathematical models have shown how heterogeneity in sexual behaviour can shape epidemiological dynamics [8,13,21] and influence the efficacy of intervention programmes [51–53]. Still, there are many consequences of host heterogeneity that are yet to be fully understood. The models introduced here and elsewhere [7,54,55] suggest that different mating strategies between the sexes can lead to considerable variation in the dynamics of STIs, but many of the evolutionary consequences of such heterogeneity are yet to be determined. For instance, Boots & Knell [56] explored a system where hosts exhibited either risky (highly active) or safe (less active) mating strategies in the presence of a sterilizing STI and found that both strategies are able to coexist for a wide range of parameters, provided the risky strategy carries a fitness benefit in the absence of disease. However, the authors did not explore how non-random mixing might affect the coexistence of risky and safe mating strategies, or whether coexistence is possible when there is a greater degree of host heterogeneity.

Simulated epidemics on SCNs are the most realistic models available for the spread of STIs in human and animal populations, but they are computationally intensive and are often difficult to parametrize. Pairwise approximations [57] offer some of the realism of SCNs by tracking the formation and break-up of partnerships, making them analytically and computationally more manageable than SCNs, but at the cost of neglecting wider population structure. Pairwise approximations are still in their infancy, but have been shown to exhibit dynamics similar to full simulations on SCNs [30,33,57]. Various degrees of host heterogeneity [57] and assortative mixing [33] have been modelled using this approach, but there is considerable scope for further research on these topics. In addition, pairwise approximations for polygynous/polyandrous mating systems are noticeably absent from the literature.

Various models incorporating heterogeneity in host contact structure have been used to study the evolution of pathogen virulence [33,39,58–60] and of antigenic diversity [61,62], but these have not been widely applied to STIs. Models have also been used to explore the role of STIs in the evolution of host-mating strategies [7,54,63], but there have been very few studies that have combined these approaches to explore coevolution between hosts and STIs. As an exception, Prado *et al.* [64] explored how host sociality (i.e. contact frequency) and pathogen virulence may coevolve on a contact network. The authors found that high levels of sociality tend to benefit more virulent pathogens, but then selection will favour more cautious hosts and subsequent reductions in virulence, which can lead to coevolutionary cycling in these traits. An exciting avenue for future work in this area would be to explicitly incorporate host and pathogen genetics within a coevolutionary framework with some plasticity in mating strategies, particularly as the predictions of such models may be amenable to testing in a wide variety of animal systems.
